# Bacterial diet influences mutation rate in *Pristionchus pacificus*

**DOI:** 10.1093/g3journal/jkag038

**Published:** 2026-02-13

**Authors:** Yinan Wang, Penghieng Theam, Shiela Pearl Quiobe, Waltraud Röseler, Hanh Witte, Christian Rödelsperger, Ralf J Sommer

**Affiliations:** Department for Integrative Evolutionary Biology, Max Planck Institute for Biology Tübingen, Max-Planck Ring 9, Tübingen 72076, Baden-Württemberg, Germany; Department for Integrative Evolutionary Biology, Max Planck Institute for Biology Tübingen, Max-Planck Ring 9, Tübingen 72076, Baden-Württemberg, Germany; Department for Integrative Evolutionary Biology, Max Planck Institute for Biology Tübingen, Max-Planck Ring 9, Tübingen 72076, Baden-Württemberg, Germany; Department for Integrative Evolutionary Biology, Max Planck Institute for Biology Tübingen, Max-Planck Ring 9, Tübingen 72076, Baden-Württemberg, Germany; Department for Integrative Evolutionary Biology, Max Planck Institute for Biology Tübingen, Max-Planck Ring 9, Tübingen 72076, Baden-Württemberg, Germany; Department for Integrative Evolutionary Biology, Max Planck Institute for Biology Tübingen, Max-Planck Ring 9, Tübingen 72076, Baden-Württemberg, Germany; Department for Integrative Evolutionary Biology, Max Planck Institute for Biology Tübingen, Max-Planck Ring 9, Tübingen 72076, Baden-Württemberg, Germany

**Keywords:** *Pristionchus pacificus*, *Caenorhabditis elegans*, mutation accumulation, mutation rate, nematodes, dietary influence

## Abstract

Mutation is a major force of evolution and its accumulation is suggested to be influenced by environmental and genetic factors in both unicellular and multicellular species. While ample of evidence showed an effect of temperature on mutation rate, the influence of diet is less well characterized, especially in multicellular organisms. Here, we present mutation accumulation (MA) rate differences for the same nematode species comparing a variety of bacterial diets. MA rates were estimated from whole-genome sequencing data of MA lines of different natural isolates of the free-living nematode *Pristionchus pacificus* on various bacterial diets isolated from *Pristionchus*-associated environments. Average single-nucleotide mutation rates varied between 1.69 × 10^−9^ and 2.23 × 10^−9^ nucleotide site^−1^ × generation^−1^, whereas the average insertion rates varied between 1.53 × 10^−10^ and 2.90 × 10^−10^ nucleotide site^−1^ × generation^−1^ and the average deletion accumulation rates varied between 3.01 × 10^−10^ nucleotide site^−1^ × generation^−1^ and 4.51 × 10^−10^ nucleotide site^−1^ × generation^−1^. We observed around a 1.4-fold mutation rate difference among groups on bacterial diets. Despite mutation-rate differences, the mutation spectra are largely unchanged. These results suggest that bacterial diet influences MA rate without drastically changing other mutational features.

## Introduction

Mutations are one of the major evolutionary forces and they are influenced by environmental and genetic factors in both unicellular and multicellular organisms (Matsuba et al. 2013; Lynch et al. 2023). Mutation accumulation (MA) experiments represent a powerful approach to study the influence of various factors on mutation rate and mutation spectra with many case studies in various organisms (Denver et al. 2012; Matsuba et al. 2013; Weller et al. 2014; Ness et al. 2015; Liu and Zhang 2019; Waldvogel and Pfenninger 2021; Lynch et al. 2023). For example, genetic factors were shown to influence mutation rate up to seven-fold in the green alga *Chlamydomonas reinhardtii* (Ness et al. 2015).

Among environmental factors potentially influencing MA, temperature has been studied in multiple animal species (Matsuba et al. 2013; Waldvogel and Pfenninger 2021). The influence of high temperature is suggested to be achieved through the increased level of stress on the organism in the nematode model organism *Caenorhabditis elegans* (Matsuba et al. 2013). In contrast to temperature, the influence of dietary conditions on MA is less studied, in particular in animals. Previous studies in *Drosophila melanogaster* suggest that poor nutritional conditions can contribute to the accumulation of deleterious mutations (Agrawal and Wang 2008). However, it is currently unclear whether diet has an influence on MA in nematodes.

In multicellular organisms, MA experiments are performed by propagating organisms for a certain number of generations, with only one or two individuals picked for establishing the next generation (Denver et al. 2012; Matsuba et al. 2013; Weller et al. 2014; Waldvogel and Pfenninger 2021). Experiments are often performed in the laboratory under highly controlled environmental conditions and with ample resources to reduce competition (Denver et al. 2012; Matsuba et al. 2013; Weller et al. 2014; Waldvogel and Pfenninger 2021). Usually, MA experiments are performed in species with a short generation time and simple maintenance. Meeting those criteria, MA experiments can be performed in the nematode *Pristionchus pacificus*, a free-living species with a generation time of around four days. This species has recently been established as a model organism for studying developmental plasticity of two alternative mouth forms, potential predation on other nematodes and self-recognition largely by using the laboratory strain *P. pacificus*  PS312 (Sommer et al. 1996; Lightfoot et al. 2019; Schroeder 2021; Sommer 2025). As *P. pacificus* is often found in association with scarab beetles, a multitude of wild isolates is available for this species (McGaughran et al. 2016). In recent times, several of these wild isolates were started to be used in natural variation studies (Dardiry et al. 2023).

To investigate how diverse dietary sources contribute to MA, we used *P. pacificus* strains of different genetic backgrounds and exposed worms of one of these strains to distinct bacterial diets. In this study, we performed MA experiments of the *P. pacificus* wild isolate RSC019 on different bacteria isolated from *Pristionchus*-associated environment. Specifically, MA data were newly generated for *P. pacificus* RSC019 on 3 bacterial diets, *E. coli*  OP50, *Hafnia alvei* LRB17 (*H. alvei* LRB17), and *Agrobacterium tumefaciens* L27 (*A. tumefaciens* L27). *H. alvei* LRB17 is also known to reduce predation and life span compared to *E. coli*  OP50 in *P. pacificus* P312, while *A. tumefaciens* L27 increased the predation but did not show any influence on life span (Akduman et al. 2018, 2020). We also obtained MA data of *P. pacificus* RSC011, a wild isolate that was subject to a recent long-term environmental induction experiment, which used a similar experimental setup as MA experiments (Quiobe et al. 2025). In this experiment, *P. pacificus* RSC011 were raised on an alternative diet *Novosphingobium* sp. L76 (referred to as “*N.* sp. L76' for the rest of the manuscript) for 100 generations. *N.* sp. L76 is known to have multiple influences on the laboratory strain *P. pacificus*  PS312 including increased predatory behavior and reduced adult lifespan (Akduman et al. 2018, 2020). *N.* sp. L76 can also increase brood size, developmental speed and the probability of producing the predatory mouth form in *P. pacificus* RSC011 (Dardiry et al. 2023; Quiobe et al. 2025; Quiobe and Sommer 2025). Comparing MA properties in those newly generated data together with the MA data generated previously in *P. pacificus*  PS312 on *E. coli*  OP50 (Weller et al. 2014), we compared MA properties in *P. pacificus* from three different genetic backgrounds and raised on four bacterial diets. In total, we observed a 1.4-fold change in accumulation rate of all three types under different dietary conditions. However, between the 2 different genetic backgrounds we observed no difference in single-nucleotide variation (SNV) accumulation rate, a two-fold change in insertions accumulation and 1.4-fold in deletion accumulation.

## Materials and methods

### Generation of MA lines

All *P. pacificus* and bacterial strains were from the Sommer lab collection (McGaughran et al. 2016; Akduman et al. 2018). Except for the standard *E. coli*  OP50, bacterial strains used in this study were isolated from *Pristionchus*-associated environments (Akduman et al. 2018). Note that the original taxonomic assignments of L27 and LRB17 in Akduman et al. (2018) were revised to *A. tumefaciens* L27 and *H. alvei* LRB17 after whole genome sequencing (Akduman et al. 2018; Athanasouli et al. 2025).

To generate MA lines of *P. pacificus* RSC019, J4 progenies of a single hermaphrodite of RSC019 were grown on the standard lab food source *E. coli*  OP50 following a protocol similar to Molnar et al. (2011). For *P. pacificus* RSC019 MA lines propagated on non-*E. coli*  OP50 diets, the F1 worms were moved as J4 progenies of the same single hermaphrodite to the new diets. To remove remnants of *E. coli*  OP50 cells F1 worms were transferred to new plates five times. In each transfer, worms were let to feed on the new bacteria for at least 15 min. From F2 to F100, all MA lines were raised at 20 °C and only one hermaphroditic J4-stage individual was transferred every generation in each line. Two previous generations of each line were kept and used to rescue when necessary (due to contamination of plates or the reduction of brood size). In *P. pacificus* RSC019, 20 lines each were grown on *E. coli*  OP50, *A. tumefaciens L27*, and *H. alvei* LRB17. All lines were frozen in liquid nitrogen every 10 generations. At the start of the experiments, the parental line was also frozen.

The MA experiment in RSC011 was done as described previously (Quiobe et al. 2025). In short, to initiate the experiment, 110 clonal lines were established by isolating individual J4 larvae onto *N.* sp. L76 and 10 lines onto *E. coli*  OP50 from the complete brood of a single hermaphrodite. Each line was transferred every 4 days by a single J4 animal with fresh *N.* sp. L76 or *E. coli*  OP50, resulting in a continuous propagation for 1 year (101 generations). Populations were cryopreserved in liquid nitrogen every 10 generations (up to F100). For this study, we randomly selected 20 lines on *N.* sp. L76 and eight lines (out of the 10 original lines) on *E. coli*  OP50 after 100 generations.

### Whole-genomic sequencing (WGS) library preparation

To gain enough material for WGS for the frozen ancestral line and MA lines of *P. pacificus* RSC019, five hermaphroditic J4 individuals were picked from each line transferred to five 6-cm nematode growth medium (NGM) plates. Progenies were collected for DNA extraction after the bacteria were depleted. DNA extraction and genomic sequencing were done at the same time for all the lines of *P. pacificus* RSC019 including the ancestral line to avoid potential batch effects. WGS library preparation was done using the Monarch Genomic DNA Purification Kit. The manufacturer's protocol was followed with some adjustments for nematode tissues. Briefly, each sample was lysed with 250 µl of tissue lysis buffer and 15 µl of proteinase K at 56 °C and kept agitated at 400 rpm for 1–1.5 h. It was then centrifuged for 3 min at maximum speed and supernatant was transferred to a new Eppendorf tube. Five microliters of RNase was added to the lysate that was then lightly and thoroughly mixed and kept agitated at 400 rpm for 10 min at 56 °C. Afterward, 400 µl of gDNA binding buffer was added to the sample. After mixing thoroughly, the sample was loaded onto the gDNA purification column. This was followed by a 3-min centrifugation to bind to the purification column at 100*×g*. Then, the same was centrifuged for 1 min more at maximum speed to remove the supernatant. The washing step was repeated twice with 500 µl of gDNA wash buffer and 1-min centrifugation at maximum each time. Finally, 32 µl of pre-heated elution buffer at 60 °C was added to the sample, which was allowed to stand for 1 min at room temperature. The elution was done by centrifuging the sample at maximum speed. WGS libraries of *P. pacificus* RSC011 MA lines were also generated with Monarch® Genomic DNA Purification Kit and in a separate single batch, following the manufacturer's instructions. All sequencing of lines of *P. pacificus* RSC019 and RSC011 were conducted by Novogene. Raw WGS data for *P. pacificus*  PS312 on *E. coli*  OP50 and *P. pacificus* RSC011 on *N*. sp. L76 were obtained from a previous publication (Weller et al. 2014; Quiobe et al. 2025).

### Generation of a reference genome for RSC019

To reduce the influence of the divergence of genetic background on mutation detection, all resequencing data were mapped to the PacBio genomes of the corresponding genetic backgrounds. Specifically, the El Paco genome was used for *P. pacificus*  PS312, and the PacBio genome generated previously was used for RSC011 (Rödelsperger et al. 2017; Quiobe et al. 2025). To generate a genome assembly of the *P. pacificus* RSC019, we followed previously established protocols (Röseler et al. 2024). In short, sequencing of a PacBio library yielded 15 Gb HiFi reads that were assembled into a raw assembly by the software Canu (version 1.4) (Korean et al. 2017). This assembly was scaffolded by the software RagTag (version 2.1.0), which used the chromosome-scale *P. pacificus* assembly for the strain PS312 (version El Paco) as reference (Rödelsperger et al. 2017, Alonge et al. 2022). Evidence-based gene annotations were generated by the software PPCAC based on strain-specific RNA-seq data and the community curated *P. pacificus* gene annotations (version El Paco annotations 3) (Athanasouli et al. 2020; Rödelsperger 2021). The transcriptomic data for the RSC019 strain were generated as described previously (Sun et al. 2021). The final assembly spans 152.6Mb with an N50 value of 23.3Mb. The resulting 27,588 gene models showed a BUSCO completeness of 94.9% (version 3 with the nematode odb9 data set) (Simão et al. 2015).

### Raw data analysis

Mutation detection was performed following the GATK pipeline (version 4.2.5.0) for germline short variant discovery (SNPs + indels) (Poplin et al. 2018). Specifically, alignment of raw sequencing data to the corresponding reference genome was done with bwa mem (version 0.7.17) (Li 2013). HaplotypeCaller, GenomicsDBImport, and GenotypeGVCFs in the GATK pipeline were used separately for variant calling for each sample, further consolidate GVCFs, and the final VCFs (Poplin et al. 2018).

### Mutation sharing detection

To detect if there were oversharing among lines in the same group, we compared the number of shared alternative homozygotes loci among pairs of line in the three genetic background separately. To have a fair comparison among pairs and remove the influence of potential background heterozygosity, we only counted loci with all lines covered by more than five reads less than 30% alternative homozygotes. Results are shown in Supplementary Figs. 1–3.

### Mutation detection

Line-specific mutations following similar criteria to those previously described, including SNPs and indels, were identified as accumulated mutations (Weller et al. 2014). Specifically, accumulated mutations were defined if one meets the following criteria: (i) only one type of homozygotes genotype exists with more than one line on the focal site; (ii) the alternative homozygotes only occurred in one line with no less than five reads in total supporting the genotype (DP in the vcf file); (iii) no alternative heterozygotes were inferred in any other line in the same group; (iv) at least 10 lines of the group had the reference homozygotes with support from more than five good quality reads; (v) genotype quality of the accumulated homozygotes was larger than 20 (GQ in the vcf file); and (vi) no other linking accumulated SNVs or indels in the same line. To reduce the influence of group size on mutation detection, lines were grouped into *P. pacificus*  PS312 on *E. coli*  OP50, RSC011 on *N.* sp. L76, RSC019 on *E. coli*  OP50, RSC019 on *H. alvei* LRB17, and RSC019 on *A. tumefaciens* L27, allowing each group having animals within 19 to 22 lines. Potential cross contamination occurred among lines would reduce the number of detected accumulated mutations. Therefore, we removed PT34 in RSC019 on *A. tumefaciens* L27, PT7 in RSC019 on *E. coli*  OP50, MAN_44 in RSC011 on *N.* sp. L76, and all eight lines in the whole group of RSC011 on *E. coli*  OP50. Mutations were filtered in each group separately.

To compare the MA rate between RSC011 on the two bacterial diets, we separately grouped the eight lines from RSC011 on *E. coli*  OP50 with 19 lines of RSC011 on *N.* sp. L76 to avoid the influence the potential cross contamination among lines. The estimated mutations of the 19 lines of RSC011 on *N.* sp. L76 showed no difference indicating comparable estimated mutation rates among analysis. The estimated SNV accumulation rates were higher and deletion accumulation rate were lower in RSC011 on *E. coli*  OP50 (Supplementary Fig. 4, Supplementary Table 5).

### Calculation of mutation rate

To reduce the influence of sequencing depth on MA rate calculation, the MA rate in each line was calculated as the total number of mutations divided by the generations and length of genomic regions with more than half lines in the group having more than five reads mapped to the loci. The influences of mutations were annotated with the bcftools (version 1.15) csq function (Danecek and McCarthy 2017). Mutations were defined as coding ones if their annotation contained one of the following consequences: missense, synonymous, or stop_gained.

### Statistical test

A Wilcoxon rank sum test was applied to test if MA rates were different among groups with wilcox.test function in R, an *F*-test was applied to test if variants of MA rates were different with var.test function in R, and a fisher's exact test was applied for testing if the mutation spectra were different among groups with fisher.test function in R.

### Estimation of false positive and false negative mutation rates

To estimate the false-positive (FP) and false-negative (FN) rate for mutation detection, we employed both experimental and computational methods. Experimentally, we tested the detection of 16 randomly picked accumulated mutations from PT9 and PT10 in the group of *P. pacificus* RSC019 on *E. coli*  OP50 with Sanger sequencing. Two of those mutations were in a distance of 2 bp, so the sequenced region was largely overlapping and covered both mutations. In total, 15 regions on the genome from the two lines were tested. Information about the primers and mutation detection in those two lines can be found in Supplementary Table 7. Sanger sequencing results were transformed to fasta format using the Bio.SeqIO package in python with the first and last 50 bp sequence with potentially low quality being removed. The generated sequences in fasta format were then used as query to blast to the reference genome of *P. pacificus* RSC019 for testing the mutation detection. Both FP and FN rates in those two lines could be estimated from Sanger sequencing of those regions in both lines. The only case with a discrepancy between the Sanger sequencing result and GATK reported mutations was found in the tested region with two mutations within a 2 bp distance (mutation set number 12 in Supplementary Table 7).

To estimate FP rates computationally, we used the WGS data of the ancestral line of *P. pacificus* RSC019 (PT1) on *E. coli*  OP50. The FP rate of the ancestral line was calculated as the proportion of alternative homozygotes that were only detected in the ancestral line among all (3 out of 5,101 alternative homozygotes in the region with the potential to call mutations in the group of *P. pacificus* RSC019 on *E. coli*  OP50).

To estimate FN rates computationally, we introduced random mutations into the reference genome of all three *P. pacificus* genetic backgrounds. We calculated the FN rate of insertions and false recall rates of deletions as the proportion of inserted mutations that were not detected after applying the same mutation detection pipeline among all inserted ones in the region with enough coverage for each sample. We randomly inserted SNVs, insertions and deletions to the chromosomes of all three reference genomes, and rerun the GATK pipeline with the edited reference genome and WGS of *P. pacificus*  PS312, RSC011, and RSC019 on *E. coli*. To simplify mutation detection, we only inserted mutations in the region where there was no mutation detected within 200 bp in the flanking region. Inserted SNVs were considered as detected if the alternative homozygotes with the same changes were detected in the same loci as they were inserted. Inserted indels were considered to be detected if alternative homozygotes of mutations as the same type were detected within 5 bp upstream, since assigning indels uniquely is not always possible.

## Results

### Mutation accumulation experiments in different *P. pacificus* isolates

Diet is an important environmental factor for all animals. In this study, we investigated MA in *P. pacificus* under different bacterial diets and genetic backgrounds. Specifically, MA data were generated for *P. pacificus* RSC019 on three bacterial diets and for RSC011 on two bacterial diets, propagating worms for 100 generations via single-worm descend under laboratory conditions. The *P. pacificus* RSC019 data is described here for the first time, whereas the *P. pacificus* RSC011 lines on *E. coli*  OP50 and *N.* sp. L76 were previously generated in a long-term environmental induction experiment in the context of developmental plasticity. The whole-genome sequencing data for the RSC011 lines on *N.* sp. L76 had been characterized previously (Quiobe et al. 2025), whereas the corresponding data on *E. coli*  OP50 is described here for the first time. In total, 20 lines of *P. pacificus* RSC011 on *N.* sp. L76, 8 on *E. coli*  OP50, and 60 lines of RSC019 separately on *E. coli*  OP50, *H. alvei* LRB17, and *A. tumefaciens* L27 and the ancestral line of RSC019 were sequenced. Those data were combined with previously published MA data of the standard laboratory strain *P. pacificus*  PS312 on *E. coli*  OP50 propagated for 142 generations (Weller et al. 2014). To reduce the influence of difference in genetic backgrounds on mutation detection, raw sequencing data were mapped to the reference genome for the corresponding *P. pacificus* strains. Published reference genomes were used for *P. pacificus*  PS312 and RSC011 (Rödelsperger et al. 2017; Quiobe et al. 2025), while the PacBio genome assembly for RSC019 was newly generated for this study using previously established protocols (Röseler et al. 2024). Detection of mutations followed the GATK pipeline (Poplin et al. 2018). While analyzing the data, we observed pairs of samples with higher number of shared mutations, indicating potential sample swap/cross contamination during the experiment (Supplementary Figs. 1–3). Therefore, one RSC019 sample on *E. coli*  OP50, one on *A. tumefaciens* L27, one RSC011 sample on *N.* sp., and eight in RSC011 on *E. coli*  OP50 were removed from the main analyses of our study. SNVs and indels which were only detected in one line in each group were identified as accumulated mutations and used for the following analysis. In total, 2,894 SNPs and 1,138 indels were detected for 99 samples in total (Supplementary Tables 1 and 2).

### Mutation rates are largely consistent between two genetic backgrounds

**Fig. 1.**
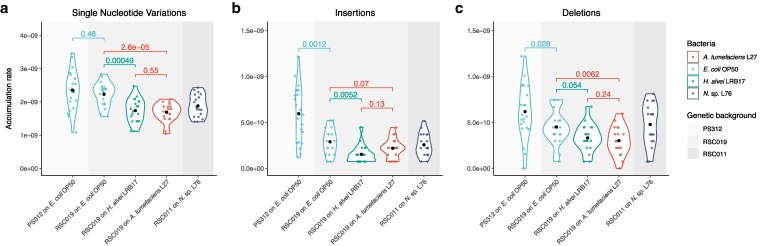
Summary of mutation accumulation rates across different conditions. Accumulation rates of a) single-nucleotide variations (SNVs), b) insertions, and c) deletions. The color in the background indicates the genetic background of each group, and the color indicates the bacterial diet. Unit of y-axis is nucleotide site^−1^ × generation^−1^. *P*-values are shown for each pair of comparisons, and statistical tests were done with the Wilcoxon's rank sum test.

To study the influence of genetic background on the MA, we compared the accumulation rates of SNVs and indels of groups of *P. pacificus*  PS312 and RSC019 on *E. coli*  OP50. For *P. pacificus* strain PS312, the average MA rate of SNVs is 2.35 ± 1.14 × 10^−9^ nucleotide site^−1^ × generation^−1^ with an average insertion rate of 5.95 ± 5.92 × 10^−10^ nucleotide site^−1^ × generation^−1^ and a deletion rate of 6.18 ± 5.73 × 10^−10^ nucleotide site^−1^ × generation^−1^ (Table 1). For RSC019, the MA rate of SNVs is 2.23 ± 0.70 × 10^−9^ nucleotide site^−1^ × generation^−1^ with an insertions rate of 2.90 ± 2.70 × 10^−10^ nucleotide site^−1^ × generation^−1^ and a deletion rate of 4.51 ± 3.66 × 10^−10^ nucleotide site^−1^ × generation^−1^ (Table 1). Thus, we observed differences in MA rates for insertions and deletions but not for SNVs between these two groups (Fig. 1a, Wilcoxon rank sum tests, Supplementary Table 4). The variance of mutation rates of *P. pacificus*  PS312 raised on *E. coli*  OP50 is higher than RSC019 on the same bacteria for insertions as well as SNVs but not deletions (Supplementary Table 4, two-sided *F*-test).

**Table 1.** Statistics of the number of mutations and properties of accumulated mutations in each mutation accumulation groups.

Strain	Bacteria	SNV	Insertion	Deletion	ts/tv	Coding/Noncoding	Genic/Intergenic	ATBias	uSNV (×10^−9^)	uInsertion (×10^−10^)	uDeletion (×10^−10^)
PS312	*E. coli* OP50	944	243	253	0.79	0.43	2.59	5.60	2.35 ± 1.14	5.95 ± 0.92	6.18 ± 5.73
RSC011	N. sp. L76	484	66	123	1.03	0.34	2.72	3.30	1.88 ± 0.65	2.57 ± 2.43	4.78 ± 4.42
RSC019	*A. tumefaciens* L27	431	56	77	1.30	0.41	1.99	4.66	1.69 ± 0.55	2.19 ± 2.09	3.01 ± 3.01
RSC019	*E. coli* OP50	569	74	115	1.21	0.41	2.16	5.24	2.23 ± 0.70	2.90 ± 2.70	4.51 ± 3.66
RSC019	*H. alvei* LRB17	466	41	89	0.96	0.39	1.93	5.24	1.74 ± 0.69	1.53 ± 1.87	3.32 ± 2.86

Note: The unit of mutation rates are per-site, per-generation. 95% confidence of mutation rates are shown in the table.

**Fig. 2.**
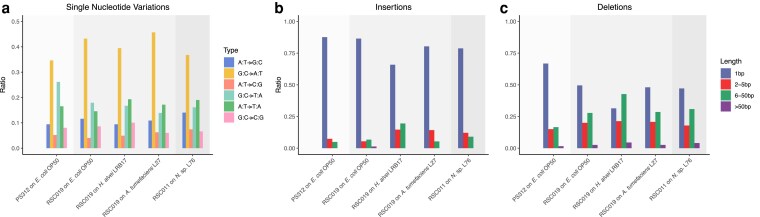
Summary of the mutation spectra across different conditions. a) Spectra of SNV and length distribution of b) insertion and c) deletions of each group. Color in the background indicates the genetic background, and color corresponds to the types of mutations.

We further tested differences in other properties of those three types of mutations. We found a significant difference in mutation spectrum between *P. pacificus*  PS312 and RSC019 (*P*-value = 0.015, Fisher's exact test, Fig. 2, Supplementary Table 4). The ratios of transitions and transversions were also significantly different between *P. pacificus*  PS312 and RSC019 (*P*-value = 5 × 10^−5^, Fisher's exact test, Supplementary Table 4), whereas the distribution of mutations across coding and noncoding regions and genic and intergenic regions were not (*P*-value > 0.05, Fisher's exact test, Supplementary Table 4). Length distribution of deletions but not insertions in *P. pacificus*  PS312 was different from that in *P. pacificus* RSC019 (*P*-value < 0.05 with Fisher's exact test, 5.1 bp for PS312 and 8.1 bp for RSC019, Supplementary Tables 3 and 4). These results show that the indel properties also had subtle changes.

### Mutation accumulation rate changed with bacterial diet

To study the effect of bacterial diet on the mutation rate, we compared the MA rates of *P. pacificus* RSC019 on different bacteria (average MA rates on *E. coli*  OP50: SNV: 2.23 ± 0.70 × 10^−9^ nucleotide site^−1^ × generation^−1^, insertion: 2.90 ± 2.70 × 10^−10^ nucleotide site^−1^ × generation^−1^, deletion: 4.51 ± 3.66 × 10^−10^ nucleotide site^−1^ × generation^−1^, on *A. tumefaciens* L27: SNV: 1.69 ± 0.55 × 10^−9^ nucleotide site^−1^ × generation^−1^, insertion: 2.19 ± 2.09 × 10^−10^ nucleotide site^−1^ × generation^−1^, deletion: 3.01 ± 3.01 × 10^−10^ nucleotide site^−1^ × generation^−1^, and on *H. alvei* LRB17: SNV: 1.74 ± 0.70 × 10^−9^ nucleotide site^−1^ × generation^−1^, insertion: 1.53 ± 1.87 × 10^−10^ nucleotide site^−1^ × generation^−1^, deletion: 3.32 ± 2.86 × 10^−10^ nucleotide site^−1^ × generation^−1^, Table 1). In *P. pacificus* RSC019, the SNV accumulation rate of worms grown on *E. coli*  OP50 was detected to be higher than those on the other two bacterial diets (Fig. 1a, Table 1, *P*-value < 0.05 compared to RSC019 on *A. tumefaciens* L27 and *H. alvei* LRB17, Wilcoxon rank sum test, Supplementary Table 4). The insertion accumulation rates of *P. pacificus* RSC019 on *E. coli*  OP50 showed significantly higher accumulation rates of insertions compared to those on *H. alvei* LRB17, and higher deletion accumulation rates compared to *A. tumefaciens* L27 (Fig. 1, b and c, Table 1). The length distributions of insertions and deletions were detected to be different between *P. pacificus* RSC019 on *E. coli*  OP50 and those on *H. alvei* LRB17 (*P*-value < 0.05, Fisher's exact test, 2.8 and 8.1 bp for *E. coli*  OP50 group and 4.4 and 10.8 bp for *H. alvei* LRB17 group, Supplementary Table 4), but not between the other two groups. The difference in mutation rate between diets is also supported by a separate comparison of our RSC011 data on *N.* sp. L76 and *E. coli*  OP50 (Supplementary Fig. 4, Supplementary Table 5). Thus, while the SNV mutation rates are largely consistent across genetic backgrounds, we observe an up to 1.3-fold increase in mutation rates between bacterial diets.

### Estimation of false positive and false negative mutation detection with GATK

Finally, we wanted to validate the quality of our mutation detection by performing false-positive (FP) and false-negative (FN) analyses. For estimating the FP and FN rates, we employed both experimental and computational strategies. Experimentally, we estimated FP and FN rates within two lines (PT9 and PT10) in the group of *P. pacificus* RSC019 on *E. coli*  OP50 as described in more details in Materials and Methods. We investigated mutation detection in both lines in 15 regions covering six SNVs, six insertions and four deletions Sanger sequencing (Supplementary Table 6). All mutations were detected as originally reported except for two closely located indels where the discrepancy in results could be attributed to the ambiguity in indel annotation. Therefore, we did not consider that specific case as FN. Given that this experimental validation did not identify any FP or FN, we would estimate the associated FP and FN rates to be close to 0%.

In addition, we computationally estimated the FP and FN rates in all three *P. pacificus* genetic backgrounds. We randomly inserted around 400 SNVs, around 3,000 insertions and deletions into the reference genome of those three genetic backgrounds and tested the detection of this set of mutations with those edited reference genome in around 20 lines from each genetic background (Supplementary Table 7). The FN rates were defined as the ratio of simulated mutations that could not be detected in the regions covered by sufficient reads in each line. We observed FN rates varied within 0–5.8% for SNVs, and false recall rates of 3.9–17.2% for insertions and 2.7–9.1% for deletions among lines (Supplementary Table 7), and estimated lower number of FN rates for insertions and deletions. To estimate the FP rates, we incorporated WGS data of the ancestral line of RSC019 (PT1), the only sequenced ancestral line among all three groups. The FP rate of the ancestral line was 0.06% and was calculated as the proportion of alternative homozygotes that were only detected in the ancestral line among all when comparing to the group of *P. pacificus* RSC019 (Supplementary Table 7). Thus, the experimental validation and computational evaluation points toward low error rates of our mutation detection procedure.

## Discussion

In this study, we compared SNV and indel accumulation rates of *P. pacificus* nematodes between genetic backgrounds and various bacterial diets. The average SNV accumulation rates observed in this study (1.08–3.34 × 10^−9^ nucleotide site^−1^ × generation^−1^) span a similar range with previously reported rates in two free-living nematodes of the genus *Caenorhabditis* (0.78–2.14 × 10^−9^ nucleotide site^−1^ × generation^−1^) (Denver et al. 2012). When further split SNVs based on their mutation and sequence context, we observed similar trends of an excess of mutations between T and A under the sequence context of TTA or TAA between *P. pacificus* groups and *C. elegans*  N2 (Supplementary Fig. 5, Konrad et al. 2019). Thus, our results suggest that the magnitude of mutation rates and fold changes appears to be conserved across the two nematode genera *Caenorhabditis* and *Pristionchus*, which were recently estimated to have diverged roughly 300 MYA (Qing et al. 2025). This indicates that mutation rates and spectra can overall be stable during nematode evolution, and we therefore speculate that these values can serve as a first estimate of mutation rate even for more distantly related free-living nematodes.

While we could not detect significant differences in the average SNV accumulation rates between genetic backgrounds, we observed significant differences in the mean and variance of indel frequencies between PS312 and RSC019. However, we would like to point out that our data sets for PS312 and RSC019 on *E. coli* are not completely comparable, as both experiments have not been carried out for the same number of generations (142 generations in the case of PS312 vs 100 generations for RSC019) and previous comparisons of first-order and second-order MA lines have identified significant differences in indel frequencies between both regimes of MA lines pointing toward the evolvability of the mutational process (Saxena et al. 2019). Thus, we cannot completely rule out an effect of such a process on the mean and variance of the observed results.

Previous studies have indicated that organisms under stressful conditions such as high temperature or poor diet quality resulting in lower fecundity or body mass can experience increased mutation rate (Agrawal and Wang 2008; Sharp and Agrawal 2012; Matsuba et al. 2013; Waldvogel and Pfenninger 2021). In our study, we showed, to the best of our knowledge for the first time, that diet can influence the MA in nematodes. One potential explanation for the difference in MA rate is the physiological stress caused by diet. Among all tested bacteria, *N*. sp. L76 and *H. alvei* LRB17 could potentially cause stress responses in *P. pacificus*  PS312, since they induced reduced adult survival compared to the other tested bacteria (Akduman et al. 2018). In contrast, *P. pacificus* RSC019 on *H. alvei* LRB17 had a slightly lower mutation rate compared to that on *E. coli*  OP50. Thus, the biological processes that affect mutation rates across different microbiota remain to be elucidated.

## Supplementary Material

jkag038_Supplementary_Data

## Data Availability

The Pacbio genome assembly of *P. pacificus* RSC019 and WGS data for *P. pacificus* RSC011 on *E. coli*  OP50 and all *P. pacificus* RSC019 are newly generated for this study and available on European Nucleotide Archive under the study accession PRJEB94012. The RSC019 genome assembly and gene annotations are available at http://www.pristionchus.org. All strains used in this study area available upon request from the corresponding author. Supplemental material available at G3 online.

## References

[jkag038-B1] Agrawal AF, Wang AD. 2008. Increased transmission of tutations by low-condition females: evidence for condition-dependent DNA repair. PLoS Biol. 6:e30. 10.1371/journal.pbio.0060030. 18271627 PMC2235904

[jkag038-B2] Akduman N et al 2020. Bacterial vitamin B12 production enhances nematode predatory behavior. ISME J. 14:1494–1507. 10.1038/s41396-020-0626-2. 32152389 PMC7242318

[jkag038-B3] Akduman N, Rödelsperger C, Sommer RJ. 2018. Culture-based analysis of *Pristionchus*-associated microbiota from beetles and figs for studying nematode-bacterial interactions. PLoS One. 13:e0198018. 10.1371/journal.pone.0198018. 29864131 PMC5986141

[jkag038-B4] Alonge M, et al 2022. Automated assembly scaffolding using RagTag elevates a new tomato system for high-throughput genome editing. Genome Biol. 23:258. 10.1186/s13059-022-02823-7. 36522651 PMC9753292

[jkag038-B5] Athanasouli M et al 2020. Comparative genomics and community curation further improve gene annotations in the nematode *Pristionchus pacificus*. BMC Genomics. 21:708. 10.1186/s12864-020-07100-0. 33045985 PMC7552371

[jkag038-B6] Athanasouli M, Loschko T, Rödelsperger C. 2025. Interspecies systems biology links bacterial metabolic pathways to nematode gene expression, chemotaxis behavior, and survival. Genome Res. 35:2363–2374. 10.1101/gr.280848.125. 40764054 PMC12487815

[jkag038-B7] Danecek P, McCarthy SA. 2017. BCFtools/csq: haplotype-aware variant consequences. Bioinformatics. 33:2037–2039. 10.1093/bioinformatics/btx100. 28205675 PMC5870570

[jkag038-B8] Dardiry M, Piskobulu V, Kalirad A, Sommer RJ. 2023. Experimental and theoretical support for costs of plasticity and phenotype in a nematode cannibalistic trait. Evol Lett. 7:48–57. 10.1093/evlett/qrac001. 37065436 PMC10091500

[jkag038-B9] Denver DR et al 2012. Variation in base-substitution mutation in experimental and natural lineages of *Caenorhabditis* Nematodes. Genome Biol Evol. 4:513–522. 10.1093/gbe/evs028. 22436997 PMC3342874

[jkag038-B10] Konrad A, Brady MJ, Bergthorsson U, Katju V. 2019. Mutational landscape of spontaneous base substitutions and small indels in experimental *Caenorhabditis elegans* populations of differing size. Genetics. 212:837–854. 10.1534/genetics.119.302054. 31110155 PMC6614903

[jkag038-B11] Koren S, et al 2017. Canu: scalable and accurate long-read assembly via adaptive k-mer weighting and repeat separation. Genome Res. 27:722–736. 10.1101/gr.215087.116. 28298431 PMC5411767

[jkag038-B12] Li H . 2013. Aligning sequence reads, clone sequences and assembly contigs with BWA-MEM [preprint]. arXiv 3997. [accessed 2025 Jul 15]. http://arxiv.org/abs/1303.3997. 10.48550/arXiv.1303.3997

[jkag038-B13] Lightfoot JW et al 2019. Small peptide–mediated self-recognition prevents cannibalism in predatory nematodes. Science. 364:86–89. 10.1126/science.aav9856. 30948551

[jkag038-B14] Liu H, Zhang J. 2019. Yeast spontaneous mutation rate and spectrum vary with environment. Curr Biol. 29:1584–1591.e3. 10.1016/j.cub.2019.03.054. 31056389 PMC6529271

[jkag038-B15] Lynch M et al 2023. The divergence of mutation rates and spectra across the Tree of Life. EMBO Rep. 24:e57561. 10.15252/embr.202357561. 37615267 PMC10561183

[jkag038-B16] Matsuba C, Ostrow DG, Salomon MP, Tolani A, Baer CF. 2013. Temperature, stress and spontaneous mutation in *Caenorhabditis briggsae* and *Caenorhabditis elegans*. Biol Lett. 9:20120334. 10.1098/rsbl.2012.0334. 22875817 PMC3565477

[jkag038-B17] McGaughran A et al 2016. Genomic profiles of diversification and enotype-phenotype association in island nematode lineages. Mol Biol Evol. 33:2257–2272. 10.1093/molbev/msw093. 27189551

[jkag038-B18] Molnar RI, Bartelmes G, Dinkelacker I, Witte H, Sommer RJ. 2011. Mutation rates and intraspecific divergence of the mitochondrial genome of Pristionchus pacificus. Mol Biol Evol. 28:2317–2326. 10.1093/molbev/msr057. 21368317

[jkag038-B19] Ness RW et al 2015. Extensive de novo mutation rate variation between individuals and across the genome of *Chlamydomonas reinhardtii*. Genome Res. 25:1739–1749. 10.1101/gr.191494.115. 26260971 PMC4617969

[jkag038-B20] Poplin R et al 2018. Scaling accurate genetic variant discovery to tens of thousands of samples [preprint]. bioRxiv 201178. [accessed 2024 Apr 3]. https://www.biorxiv.org/content/10.1101/201178v3. 10.1101/201178

[jkag038-B21] Qing X et al 2025. Phylogenomic insights into the evolution and origin of *Nematoda*. Syst Biol. 74:349–358. 10.1093/sysbio/syae073. 39737664

[jkag038-B22] Quiobe SP et al 2025. EBAX-1/ZSWIM8 destabilizes miRNAs, resulting in transgenerational inheritance of a predatory trait. Sci Adv. 11:eadu0875. 10.1126/sciadv.adu0875. 40073139 PMC11900880

[jkag038-B23] Quiobe SP, Sommer RJ. 2025. Adaptive consequences of transgenerational inheritance of a predatory mouth-form trait in nematodes [preprint]. bioRxiv 680509. [accessed 2025 Nov 2]. https://www.biorxiv.org/content/10.1101/2025.10.05.680509v1. 10.1101/2025.10.05.680509

[jkag038-B24] Rödelsperger C et al 2017. Single-molecule sequencing reveals the chromosome-scale genomic architecture of the nematode model organism *Pristionchus pacificus*. Cell Rep. 21:834–844. 10.1016/j.celrep.2017.09.077. 29045848

[jkag038-B25] Rödelsperger C . 2021. The community-curated *Pristionchus pacificus* genome facilitates automated gene annotation improvement in related nematodes. BMC Genomics. 22:216. 10.1186/s12864-021-07529-x. 33765927 PMC7992802

[jkag038-B26] Röseler W, Sommer RJ, Rödelsperger C. 2024. Nematode genome announcement: a chromosome-scale genome assembly for the *Pristionchus pacificus* reference mapping strain PS1843. J Nematol. 56:20240063. 10.2478/jofnem-2024-0063. 39290648 PMC11406906

[jkag038-B27] Saxena AS, Salomon MP, Matsuba C, Yeh S-D, Baer CF. 2019. Evolution of the mutational process under relaxed selection in *Caenorhabditis elegans*. Mol Biol Evol. 36:239–251. 10.1093/molbev/msy213. 30445510 PMC6367967

[jkag038-B28] Schroeder NE . 2021. Introduction to *Pristionchus pacificus* anatomy. J Nematol. 53:e2021–e2091. 10.21307/jofnem-2021-091. PMC8574906 34761228

[jkag038-B29] Sharp NP, Agrawal AF. 2012. Evidence for elevated mutation rates in low-quality genotypes. Proc Natl Acad Sci U S A. 109:6142–6146. 10.1073/pnas.1118918109. 22451943 PMC3341077

[jkag038-B30] Simão FA, Waterhouse RM, Ioannidis P, Kriventseva EV, Zdobnov EM. 2015. BUSCO: assessing genome assembly and annotation completeness with single-copy orthologs. Bioinformatics. 31:3210–3212. 10.1093/bioinformatics/btv35. 26059717

[jkag038-B31] Sommer RJ . 2025. *Pristionchus*—beetle associations: towards a new natural history. J Invertebr Pathol. 209:108243. 10.1016/j.jip.2024.108243. 39644992

[jkag038-B32] Sommer RJ, Carta L, Kim S-Y, Sternberg PW. 1996. Morphological, genetic and molecular description of *Pristionchus pacificus* sp. n. (Nematoda: *Neodiplogastridae*). Fundament Appl Nematol. 19:511–521.

[jkag038-B33] Sun S, Rödelsperger C, Sommer RJ. 2021. Single worm transcriptomics identifies a developmental core network of oscillating genes with deep conservation across nematodes. Genome Res. 31:1590–1601. 10.1101/gr.275303.121. 34301622 PMC8415380

[jkag038-B34] Waldvogel A-M, Pfenninger M. 2021. Temperature dependence of spontaneous mutation rates. Genome Res. 31:1582–1589. 10.1101/gr.275168.120. 34301628 PMC8415371

[jkag038-B35] Weller AM, Rödelsperger C, Eberhardt G, Molnar RI, Sommer RJ. 2014. Opposing forces of A/T-biased mutations and G/C-biased gene conversions shape the genome of the nematode *Pristionchus pacificus*. Genetics. 196:1145–1152. 10.1534/genetics.113.159863. 24414549 PMC3982703

