# Correction to: Bacterial diet influences mutation rate in *Pristionchus pacificus*

**DOI:** 10.1093/g3journal/jkag145

**Published:** 2026-06-17

This is a correction to: Yinan Wang, Penghieng Theam, Shiela Pearl Quiobe, Waltraud Röseler, Hanh Witte, Christian Rödelsperger, Ralf J Sommer, Bacterial diet influences mutation rate in *Pristionchus pacificus*, *G3 Genes|Genomes|Genetics*, Volume 16, Issue 4, April 2026, jkag038, https://doi.org/10.1093/g3journal/jkag038

In the originally published version of this manuscript, Figure 2 incorrectly duplicated Figure 1.

This error has been corrected, and the manuscript has been updated with the correct version of Figure 2.

