# Supplementary material for: Bacterial diet influences mutation rate in Pristionchus pacificus
Source: G3 (Bethesda). 2026 Feb 13;16(4):jkag038. doi: 10.1093/g3journal/jkag038 (PMC13042309; doi:10.1093/g3journal/jkag038)
Supplement: jkag038_Supplementary_Data [file jkag038_supplementary_data.zip › Supplemental_Material_G3-2025-406371.docx]

**Supplementary Material**

**Supplementary Figure 1:** Number of shared alternative homozygotes in pairs in PS312 on *E. coli* OP50. Results indicate little mutation sharing among lines.

**Supplementary Figure 2:** Number of shared alternative homozygotes in pairs in RSC019. Results indicate large proportion of mutation sharing between PT33 and PT34, and mild mutation sharing between PT6 and PT7.

**Supplementary Figure 3:** Number of shared alternative homozygotes in pairs in RSC011. Results indicate mild mutation sharing between MAN_35 and MAN_44, and large proportion of mutation sharing among MAE lines (RSC011 on *E. coli* OP50).

**Supplementary Figure 4:** Mutation rate comparison between RSC011 on *E. coli* OP50 and *N.* sp. L76. Each line of RSC011 on *E. coli* OP50 were group with the group on *N.* sp. L76 separately for accumulated mutation detection and mutation rates calculation. Mutation rates of RSC011 on *N.* sp. L76 were also estimated in these eight groups and shown.

**Supplementary Figure 5:** **Mutational spectra across 96 SNV types in *P. pacificus* and *C. elegans* N2**. SNVs are divided into 96 types based on the mutation and the nucleotide 1 bp up and down stream of it. a-f) mutation spectra of *P. pacificus* groups and g) mutation spectra of *C. elegans* N2.

**Supplementary Table 1:** Summary information of all mutations.

**Supplementary Table 2:** Summary information of all lines.

**Supplementary Table 3:** Full list of statistics of mutations in all five groups.

**Supplementary Table 4:** *p*-values of comparisons of mutation properties among groups.

**Supplementary Table 5:** Mutation rates estimated for RSC011 on *E. coli* OP50 and on *N.* sp. L76.

**Supplementary Table 6**: Primers used for sanger sequencing.

**Supplementary Table 7:** Computational estimation of FN rates.
