# Supplementary figures and images for: Bacterial diet influences mutation rate in Pristionchus pacificus
Source: G3 (Bethesda). 2026 Feb 13;16(4):jkag038. doi: 10.1093/g3journal/jkag038 (PMC13042309; doi:10.1093/g3journal/jkag038)

## Shared Alternative Homozygotes Between Samples

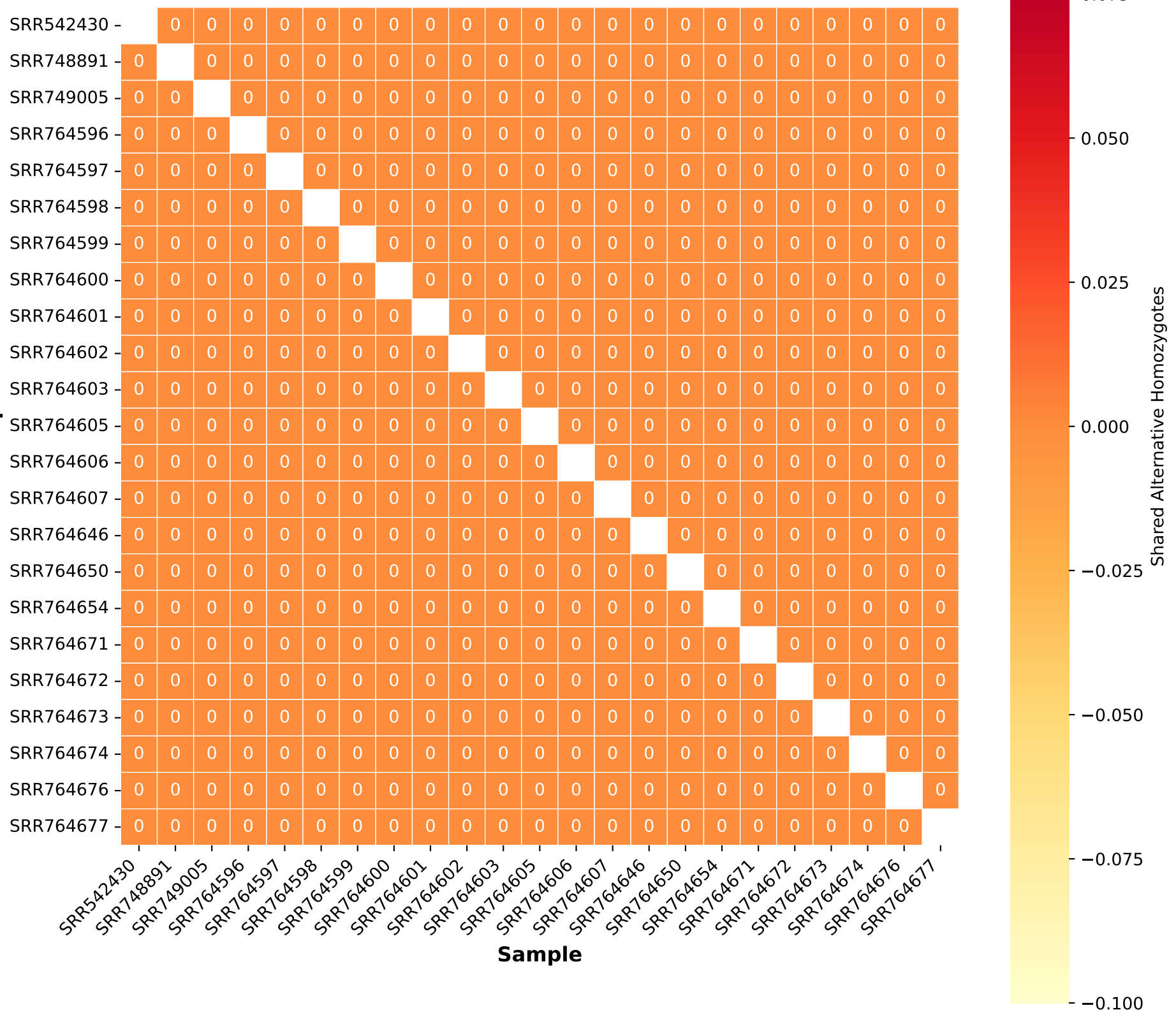

Supplement: jkag038_Supplementary_Data [file jkag038_supplementary_data.zip › Supplemental_Fig._1_G3-2025-406371.pdf]

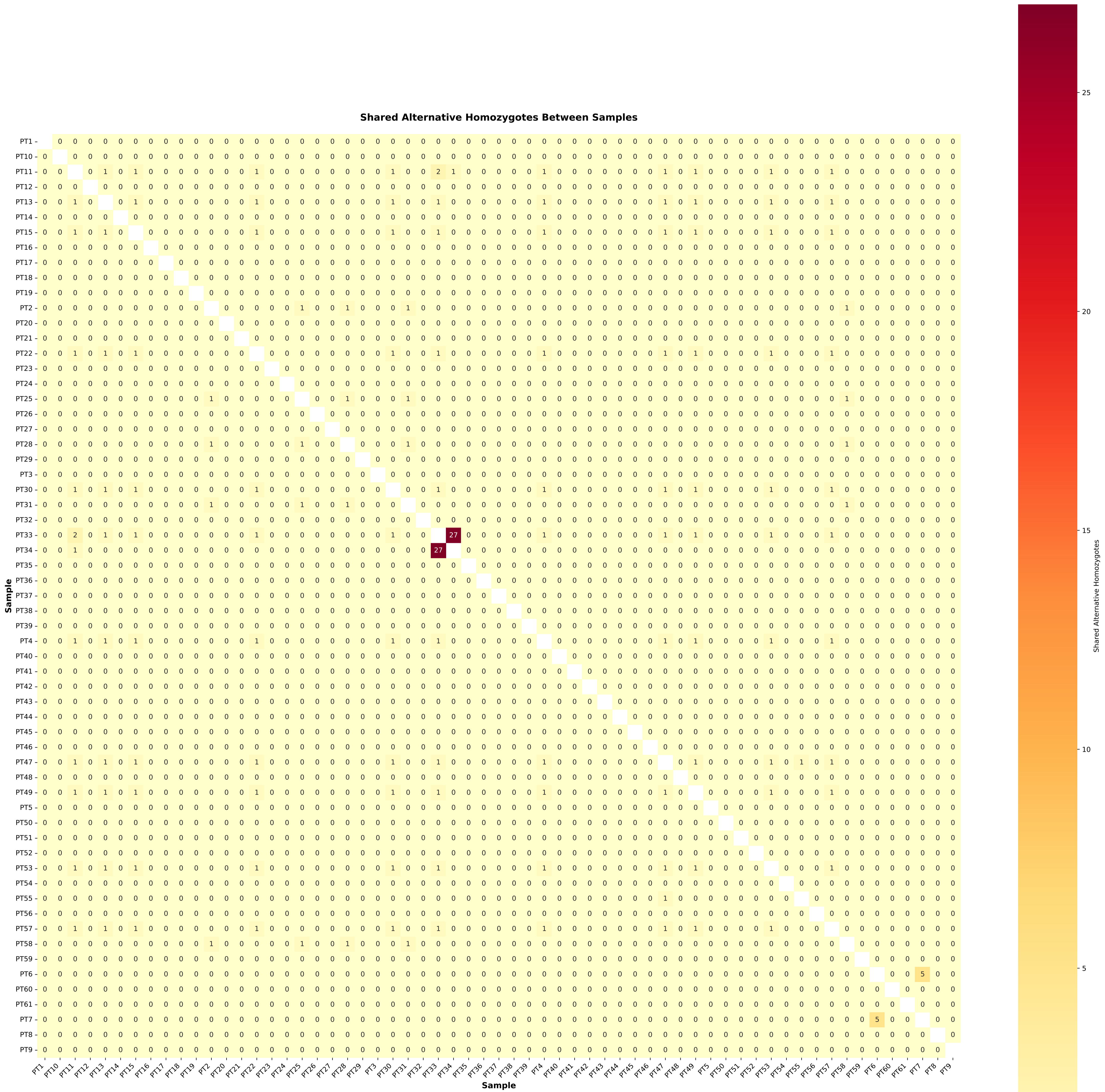

Supplement: jkag038_Supplementary_Data [file jkag038_supplementary_data.zip › Supplemental_Fig._2_G3-2025-406371.pdf]

## Shared Alternative Homozygotes Between Samples

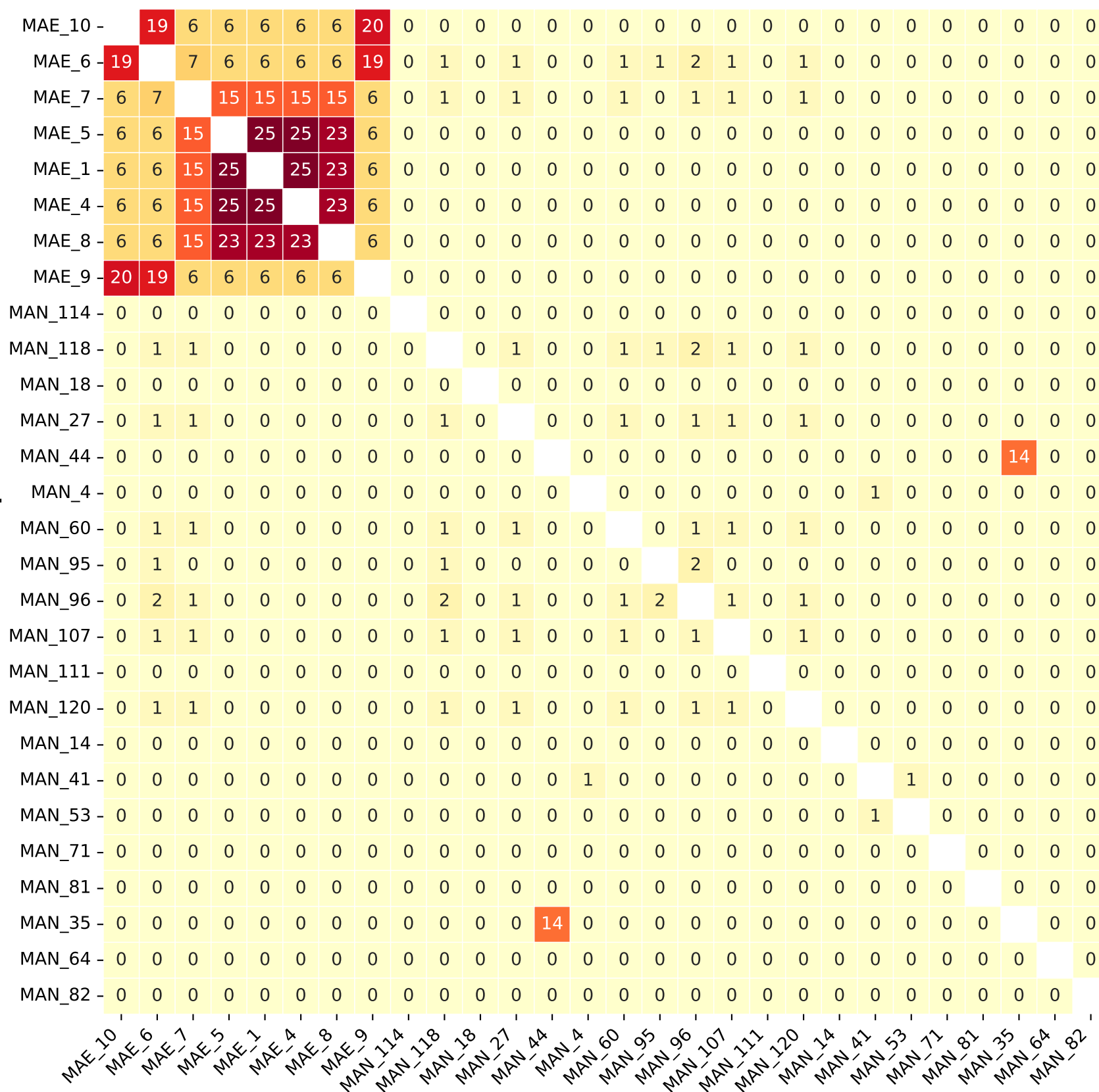

Shared Alternative Homozygotes

Supplement: jkag038_Supplementary_Data [file jkag038_supplementary_data.zip › Supplemental_Fig._3_G3-2025-406371.pdf]

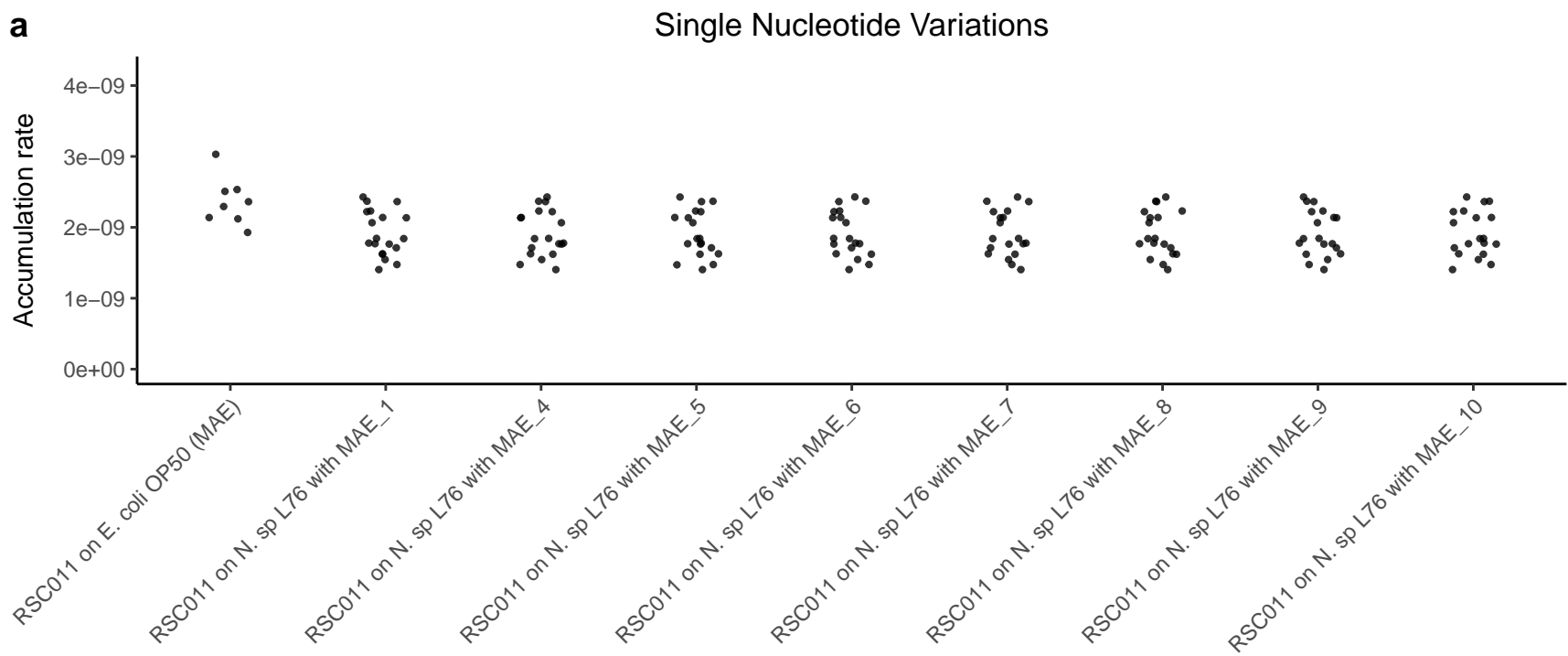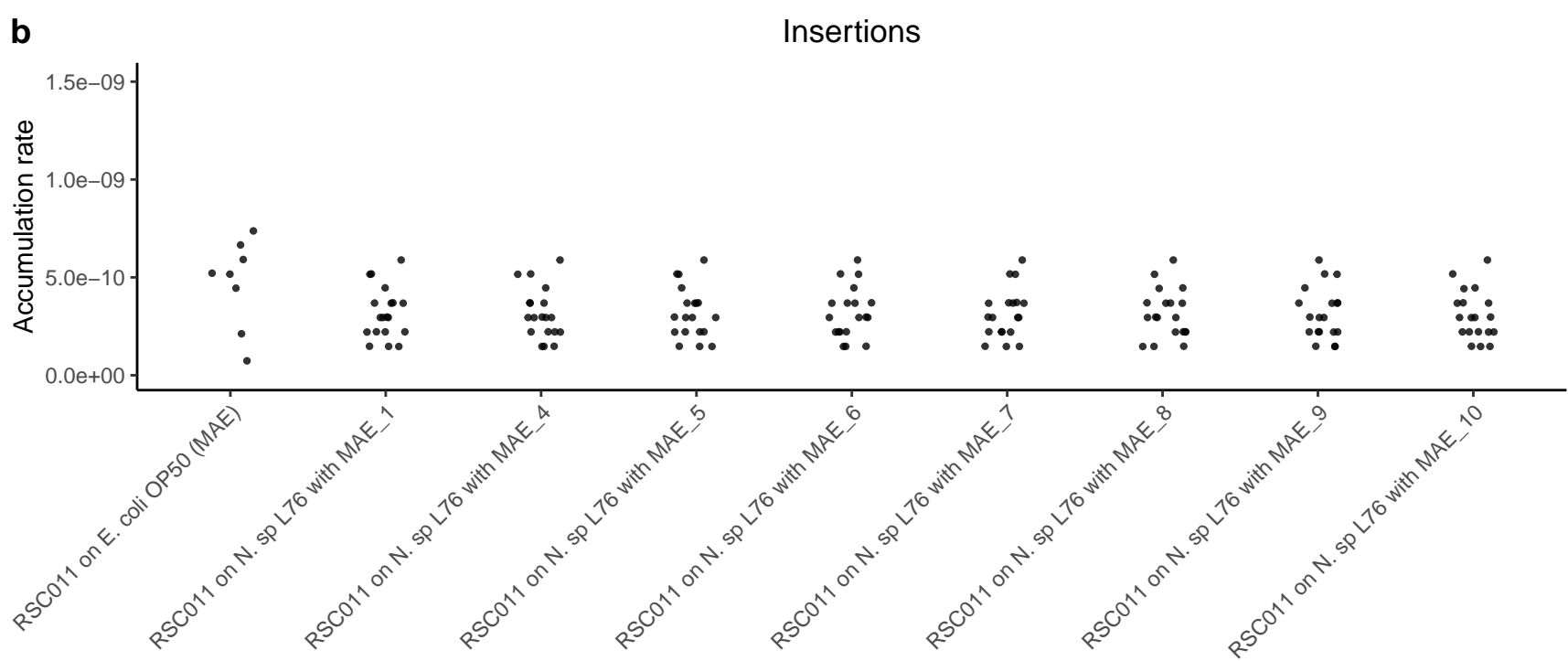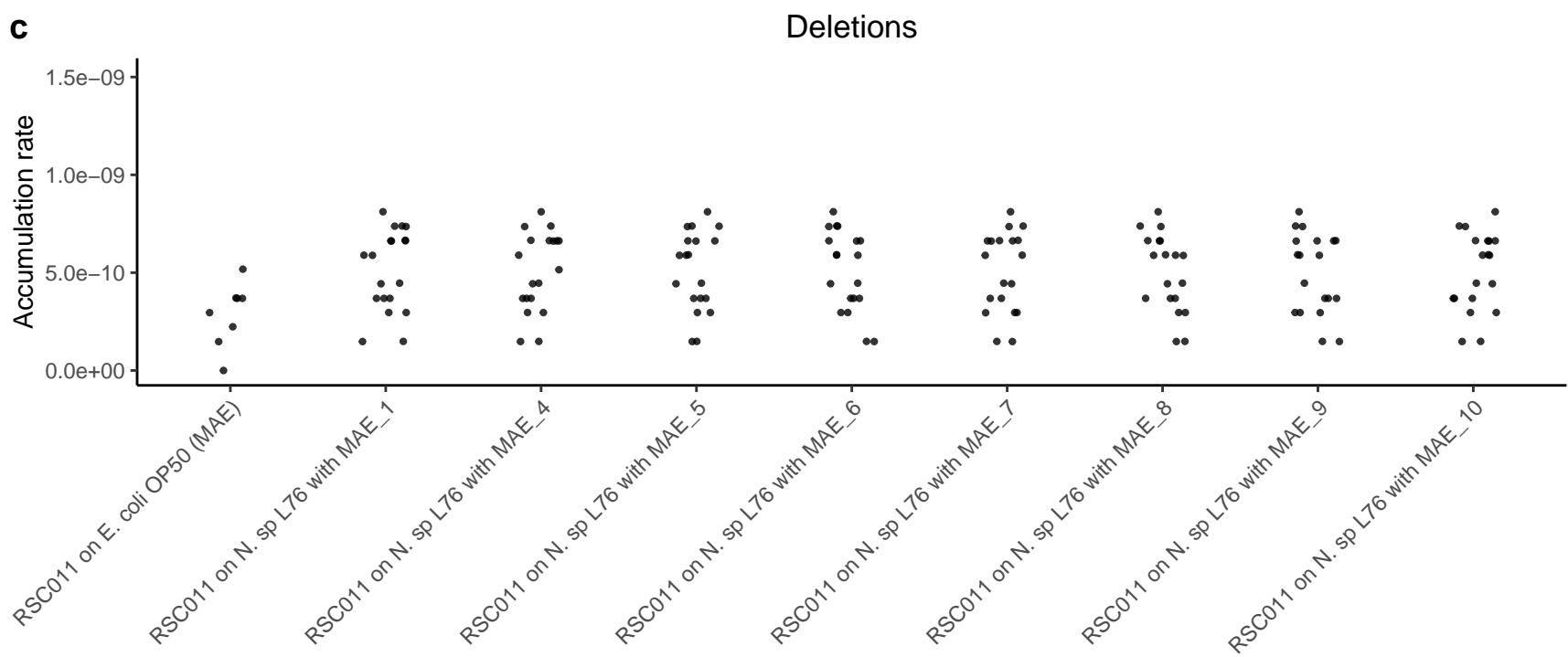

Supplement: jkag038_Supplementary_Data [file jkag038_supplementary_data.zip › Supplemental_Fig._4_G3-2025-406371.pdf]

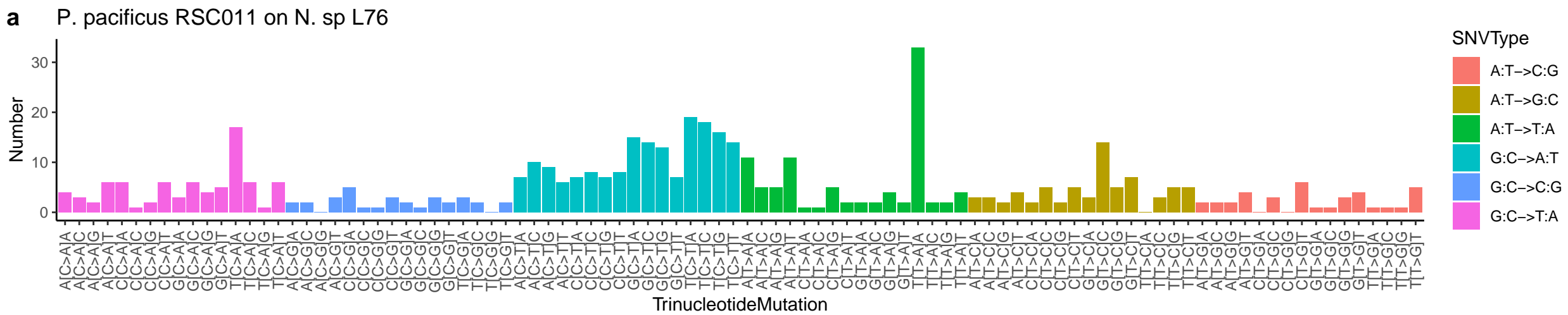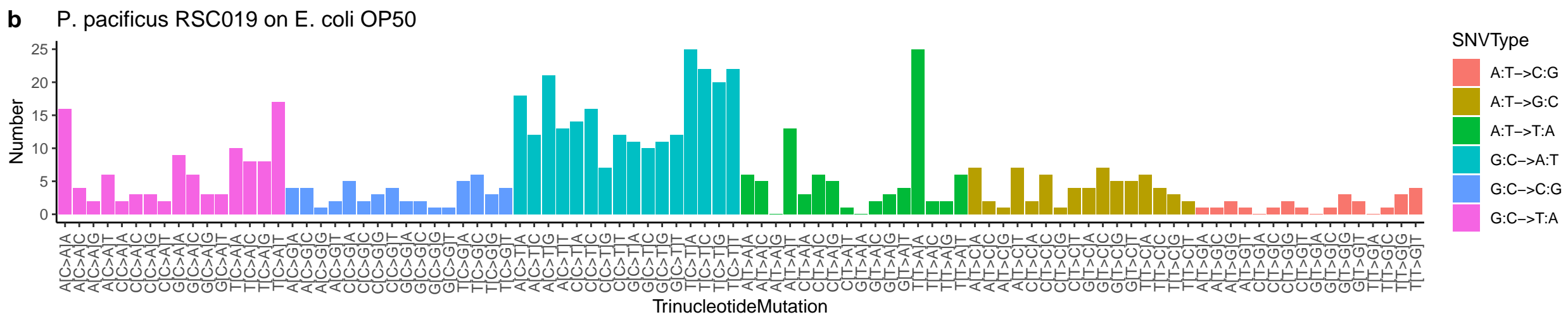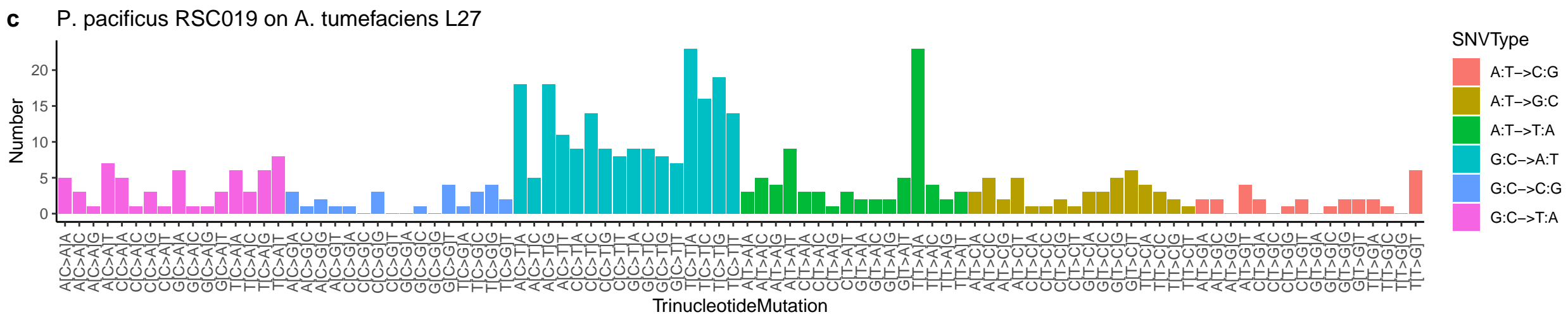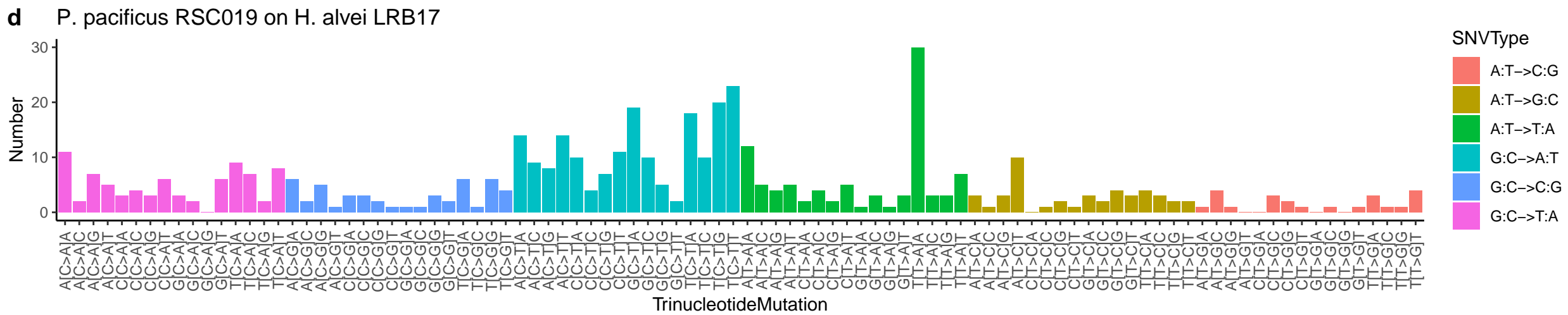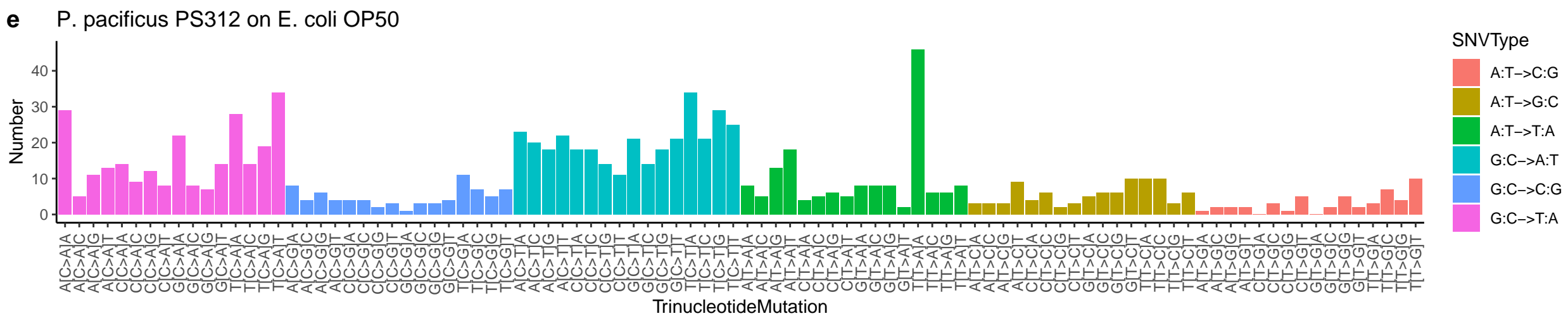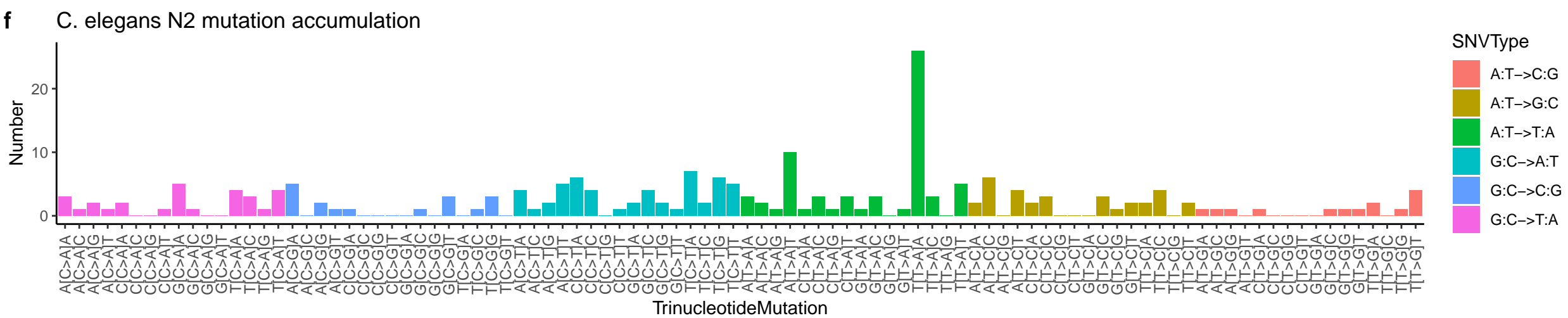

Supplement: jkag038_Supplementary_Data [file jkag038_supplementary_data.zip › Supplemental_Fig._5_G3-2025-406371.pdf]
